# A Redox-Initiated Cascade Approach to Furan-Functionalized Polyisoprene with Time-Dependent Antibacterial Activity

**DOI:** 10.3390/polym18172086

**Published:** 2026-08-28

**Authors:** Cui-Cui Wang, Jin-Hua Wang

**Affiliations:** 1 School of Pharmacy, Shandong Provincial Engineering Research Center of Novel Pharmaceutical Excipients, Sustained and Controlled Release Preparations, Dezhou University, Dezhou 253015, China; 15662408962@163.com; 2 Malaysian Institute of Chemical and Engineering Technology, University Kuala Lumpur, Lot 1988 Kawasan Perindustrian Bandar Vendor, Taboh Naning, Alor Gajah 78000, Malaysia

**Keywords:** polyisoprene, emulsion polymerization, cascade reactions, *trans*-1,4-polyisoprene, antibacterial activity

## Abstract

A novel furan-functionalized polyisoprene (Furan-PIP) was synthesized via a one-pot, redox-initiated cascade approach using tert-butyl hydroperoxide (TBHP) and tetraethylenepentamine (TEPA) as the initiating system. The polymerization of isoprene proceeded through radical emulsion polymerization, accompanied by in situ epoxidation of the resulting double bonds, followed by ring-opening and furanization reactions. The chemical structure of Furan-PIP was qualitatively characterized by FT-IR and ^1^H NMR spectroscopy. The appearance of characteristic signals at δ 3.97 and 3.37 ppm (furan ring protons) and δ 8.43 ppm (formyloxy proton) in the ^1^H NMR spectrum, together with the corresponding FT-IR absorptions at 1725 cm^−1^ (C=O) and 1015 cm^−1^ (furan ring), confirmed the successful incorporation of furan and ring-opened moieties. Residual epoxide signals were negligible, indicating near-complete consumption of epoxy groups during the cascade process. Gel permeation chromatography (GPC) revealed a high-molecular-weight polymer ($\bar{M_{n}}$ = 45,892 g/mol, PDI = 2.362). The Furan-PIP exhibited a glass transition temperature (Tg) of −49.6 °C and a single-stage thermal degradation at 312 °C. Subsequently, a pre-synthesized antibacterial zinc complex Zn(L-Cl) was physically encapsulated into the Furan-PIP matrix to fabricate a composite material. The composite exhibited time-dependent antibacterial activity against *Staphylococcus aureus* (*S. aureus*) over 48 h, leveraging the intrinsic antibacterial property of Zn(L-Cl) previously reported by our group. This work presents a simple and efficient strategy for preparing furan-functionalized elastomers with potential applications in antibacterial materials.

## 1. Introduction

Polymerization stands as one of the most transformative chemical processes, converting low-molecular-weight monomers into macromolecular materials with emergent properties distinct from their constituent units. Among the diverse polymerization mechanisms, chain-growth polymerization (CGP) dominates industrial practice, wherein active centers generated by initiators sequentially incorporate monomers to propagate polymer chains. The choice of initiation system profoundly influences not only reaction kinetics and molecular weight characteristics but also the chemical architecture of the resulting polymers, thereby determining their ultimate performance in target applications. Emulsion polymerization represents a particularly versatile implementation of CGP, dispersing hydrophobic monomers in aqueous media via surfactant stabilization [1,2]. This technique offers distinct advantages including superior heat management, controlled particle morphology, and direct applicability of the resulting latexes in coatings, adhesives, and biomedical materials [3]. However, conventional thermal initiation systems require elevated temperatures for radical generation, incurring substantial energy costs and potential safety hazards associated with thermal runaway [4]. These limitations have catalyzed intensive research into redox initiation strategies capable of operating under milder conditions [5,6].

Redox initiation systems generate active radicals through electron transfer between oxidants and reductants, achieving effective polymerization at ambient or sub-ambient temperatures with markedly reduced activation energies compared to thermal counterparts [7,8]. Since Bacon’s seminal demonstration in 1946 that specific oxidation–reduction couples could initiate vinyl polymerization [9], this technology has undergone continuous refinement. Among the diverse redox systems developed, the tert-butyl hydroperoxide/tetraethylenepentamine (TBHP/TEPA) combination has attracted particular attention due to its operational simplicity, cost-effectiveness, and versatile applicability across monomer classes [10]. The lipophilic tert-butyl group facilitates radical entry into micelles, while TEPA functions as both reductant and chain transfer agent, enabling unique kinetic control unavailable with hydrophilic persulfate systems [11,12].

Isoprene (2-methyl-1,3-butadiene), the monomeric precursor to natural rubber, presents distinctive challenges and opportunities for redox-mediated emulsion polymerization. The polymerization of this conjugated diene can proceed via multiple regio- and stereochemical pathways—1,4-addition yielding cis or trans configurations, and 1,2- or 3,4-addition generating pendant vinyl groups—giving rise to complex microstructural arrangements including varied tacticities and sequence distributions [13]. Historically, research on synthetic polyisoprene has focused predominantly on controlling these microstructures through coordination polymerization, treating polymerization as a process of structural construction alone wherein the primary objective is precise control of backbone stereoregularity [14,15].

However, accumulating evidence suggests that TBHP/TEPA-mediated isoprene polymerization exhibits a specific complexity transcending conventional chain-growth mechanisms. Spectroscopic studies by Li et al. [16] have detected oxidative by-products including epoxides and hydrofurans along the polymer backbone, implying that the TBHP/TEPA system generates not only initiating radicals (t-BuO∙) but also peroxy radicals (t-BuOO∙) capable of attacking pendant double bonds on already-formed polymer chains [17,18]. This dual radical generation transforms the process from simple chain growth into a coupled polymerization-functionalization network, wherein polymer chains are constructed and chemically modified concurrently rather than sequentially [19].

Such oxidative modification may proceed via cascade reactions including epoxidation, ring-opening, oxidation, and furanylation/esterification [20,21,22]. Critically, the coexistence of fully converted (furan-rich), partially converted (epoxy/furan mixed), and epoxide-retained topologies under varying conditions indicates a condition-dependent pathway switching phenomenon [23,24]. Traditional homogeneous kinetic models fail to explain this behavior, and quantitative correlations between reaction conditions and topological outcomes remain elusive. Whether the dual role of TBHP/TEPA—initiation versus functionalization—can be systematically controlled, and how the balance between polymerization and functionalization dictates the ultimate chemical architecture, constitute fundamental questions motivating the present investigation. The oxygen-containing functionalities generated through TBHP/TEPA-mediated polymerization—epoxy, hydroxyl, furan, and formate ester groups—provide versatile sites for subsequent material functionalization. In particular, the immobilization of bioactive metal complexes within soft polymer matrices represents a promising strategy for achieving sustained therapeutic activity [25]. Current approaches predominantly employ rigid crystalline frameworks such as Metal–Organic Frameworks (MOFs) or semi-rigid hydrogel networks, which suffer from limited mechanical compliance, poor processability, or burst release kinetics [26].

Polyisoprene rubber offers an attractive alternative due to its inherent elasticity, hydrophobicity, and established industrial relevance [10]. However, conventional post-polymerization epoxidation yields fixed oxygen contents with limited scope for systematic optimization [27]. The TBHP/TEPA redox-initiated approach enables continuous tunability of functional group type and density through formulation design, generating diverse oxygen-rich functionalities without compromising backbone integrity [16]. This synthetic versatility permits the establishment of quantitative structure-property relationships and rational optimization of host–guest interactions for specific applications.

In this work, we report the development of a redox-initiated emulsion polymerization system using TBHP/TEPA for the one-pot synthesis of a furan-functionalized polyisoprene (Furan-PIP). The TBHP/TEPA system is demonstrated to serve a dual function: it not only initiates the free-radical polymerization of isoprene but also drives a cascade of epoxidation, ring-opening, and furanization reactions, leading to the formation of a polymer backbone bearing furan rings, formyloxy groups, and residual hydroxyl/carbonyl functionalities. The structural features of the resulting Furan-PIP are comprehensively characterized by FT-IR, GPC, ^1^H NMR, DSC, DMA, and TGA, revealing a trans-1,4-polyisoprene backbone with a unique combination of molecular weight, thermal, and viscoelastic properties. Furthermore, leveraging the hydrophobic polymer matrix as a carrier, we fabricate an antibacterial composite by incorporating a tetranuclear zinc(II) Schiff base complex (Zn(L-Cl)), previously reported by our group to exhibit potent antibacterial activity against *Staphylococcus aureus* [28], into the Furan-PIP matrix. The composite exhibits time-dependent antibacterial activity, demonstrating the potential of Furan-PIP as a versatile platform for functional material applications.

## 2. Experimental Method

### 2.1. Synthesis of Furan-Functionalized Polyisoprene (Furan-PIP)

Detailed information on reagents, equipment, and characterization methods is provided in the Supporting Information (Tables S1 and S2).

**Scheme 1:**
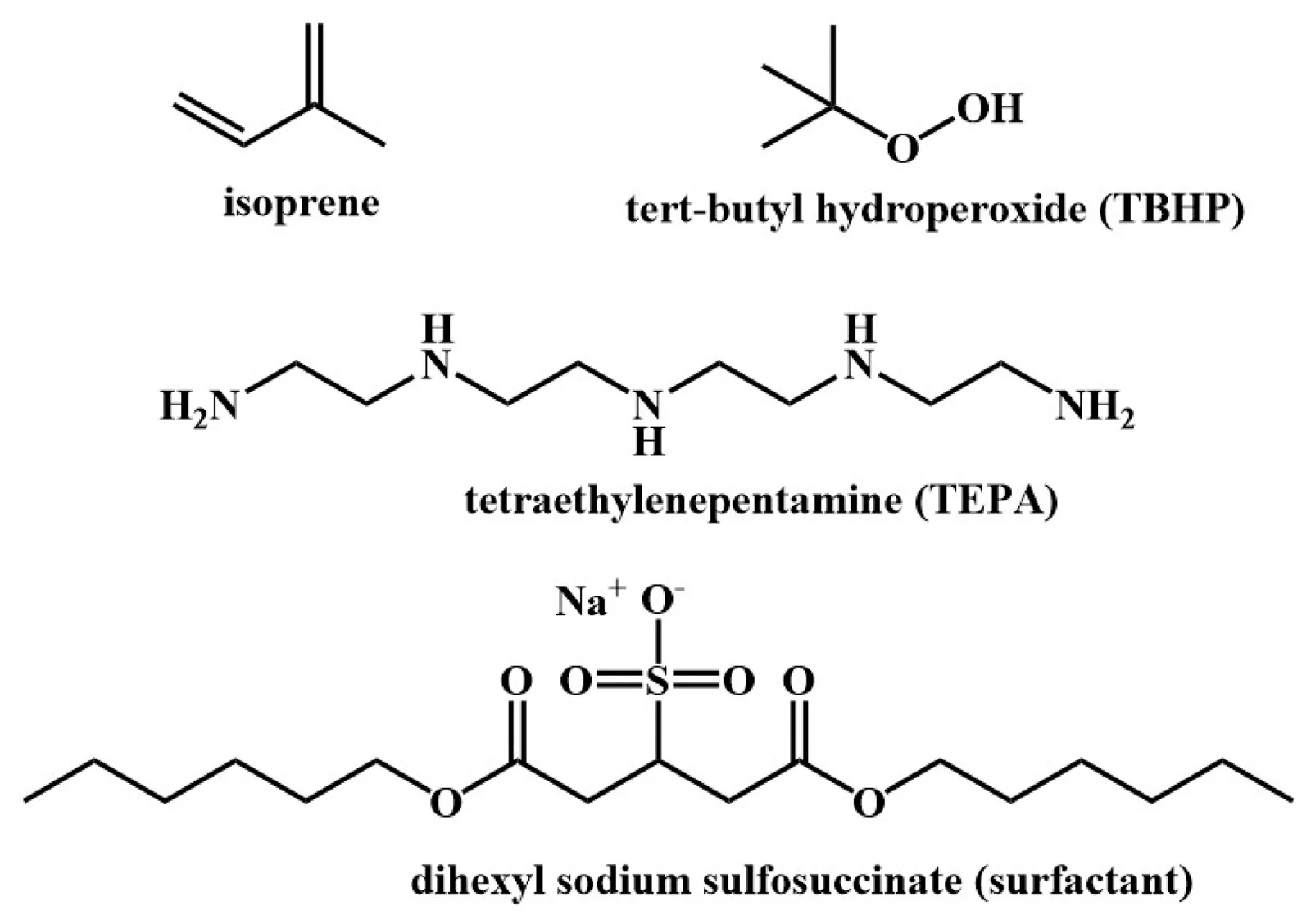
Chemical structures of the key materials used in this study.

Purify isoprene monomer with 25% aqueous NaOH in a separating funnel (Scheme 1). Pour isoprene and 25% aqueous NaOH into the separating funnel at a ratio of 1:1. Shake the mixture thoroughly. Then, let the layers separate and note the colors of both the isoprene (top) and aqueous NaOH (bottom) layers. Remove the bottom layer. Continue all the steps above until isoprene changes from yellowish to clear liquid. Add deionized water into the purified isoprene at the ratio of 1:1 and continue the steps above until the purified isoprene reaches pH = 7.

The reaction was carried out in an inert atmosphere (nitrogen) with isoprene (1.5 M), dihexyl sodium sulfosuccinate (50 mM) as the surfactant, and TBHP/TEPA (15 mM) as the redox initiator (Scheme 1). The process was performed at 25 °C with a stirring speed of 150 rpm for 48 h. The resulting products were then isolated and purified for subsequent characterization.

To ensure accuracy and reproducibility, the monomer conversion was determined gravimetrically. A culture dish (diameter: 6 cm) was weighed and recorded as m_1_. Approximately 10 g of the final product was transferred into the dish, and the total mass was recorded as m_2_. The product was washed thoroughly with methanol to remove unreacted isoprene monomer, followed by a rinse with deionized water to eliminate residual solvents and impurities. The product was then dried to a constant weight in a vacuum oven attached with vacuum pump at 30 °C for 20 h. The drying process was considered complete when the mass change between successive 30 min drying cycles was less than 5 mg. After cooling in a desiccator, the total mass of the Petri dish and the purified and dried polymer was recorded as m_3_. The degree of conversion was calculated using Equation (1). The obtained conversion was 97.57%.

(1) $$\mathrm{conversion}\;(\%)\;=\frac{\left(m_{3}-m_{1}\right)}{\left(m_{2}-m_{1}\right)}\times \frac{M_{t}}{M_{m}}\times 100\%$$

where

m_3_ − m_1_: mass of the final dried product from the sample (g).
  m_2_ − m_1_: mass of the crude sample (g).
  Mt: total mass of the synthetic product obtained (g).
  Mm: total mass of the key reactant charged (g).

### 2.2. Synthesis of Zn(L-Cl)-Furan-PIP Complex

To construct a composite material with long-lasting antibacterial activity, this study employed an in situ doping and encapsulation strategy to prepare Zn(L-Cl)-Furan-PIP. Specifically, prior to the redox-initiated emulsion polymerization of Furan-PIP as described in Section 2.1, a predetermined amount (5 wt% relative to the mass of the isoprene monomer) of the tetranuclear zinc(II)-Schiff base complex (Zn(L-Cl)), synthesized according to a method previously reported by our group [28], was directly dispersed as a functional filler into the reaction emulsion. This complex, along with the isoprene monomer, surfactant (dihexyl sodium sulfosuccinate), and the TBHP/TEPA initiator system, was subjected to polymerization under a nitrogen atmosphere at 25 °C with stirring at 150 rpm for 48 h. During this process, the TBHP/TEPA system not only initiated the polymerization of isoprene and the subsequent cascade of epoxidation, ring-opening, and furanization reactions but also facilitated physical entanglement and encapsulation of the growing polyisoprene chains with the dispersed Zn(L-Cl) crystals. This ultimately resulted in the formation of a homogeneous composite, with Zn(L-Cl) as the nanodispersed phase and the functionalized polyisoprene (Furan-PIP) as the continuous matrix. The uniform dispersion of Zn(L-Cl) within the Furan-PIP matrix was further confirmed by scanning electron microscopy–energy dispersive X-ray spectroscopy (SEM-EDS) elemental mapping (Figure S1), which revealed a homogeneous distribution of zinc across the composite surface. This observation supports the successful encapsulation of the Zn(L-Cl) complex as a nanodispersed phase within the polymer matrix and implies its structural stability under the TBHP/TEPA polymerization conditions (25 °C, N_2_ atmosphere), as no signs of degradation or aggregation were detected. Upon completion of the reaction, the product was thoroughly washed with methanol to remove unreacted monomer, rinsed with deionized water, and dried to constant weight, yielding the final Zn(L-Cl)-Furan-PIP composite material. The washing step effectively removes unreacted monomer, surfactant, and residual initiators, while the physically encapsulated Zn(L-Cl) complex is retained within the polymer matrix.

### 2.3. Thermal Characterization

Differential scanning calorimetry (DSC) was performed using a NETZSCH DSC 200 F3 (Selb, Germany). The sample was cooled to −100 °C at 20 °C/min, and subsequently heated from −100 °C to 50 °C at 20 °C/min under a nitrogen atmosphere. The glass transition temperature (*Tg*) was obtained from the heat-flow curve.

The DMA measurements were performed using a TA Instruments DMA 850 (New Castle, DE, USA) in tension mode. Specimens with dimensions of 6 cm × 1.2 cm were scanned from −100 °C to room temperature at a heating rate of 2 °C/min under a frequency of 10 Hz, and liquid nitrogen was applied as the cooling medium.

Thermogravimetric analysis (TGA) was performed to evaluate the thermal stability and degradation behavior of polymers. By monitoring sample mass upon heating, TGA provides data on decomposition temperatures, weight-loss stages and residual char content, which allows comparison of thermal resilience for polymers with different microstructures. Approximately 10 mg samples were heated from 35 °C to 850 °C at a heating rate of 10 °C/min under a nitrogen flow of 20 mL/min. Mass-loss curves were recorded and plotted as a function of temperature, and the residual weight was evaluated.

## 3. Results and Discussion

### 3.1. FT-IR

**Figure 1:**
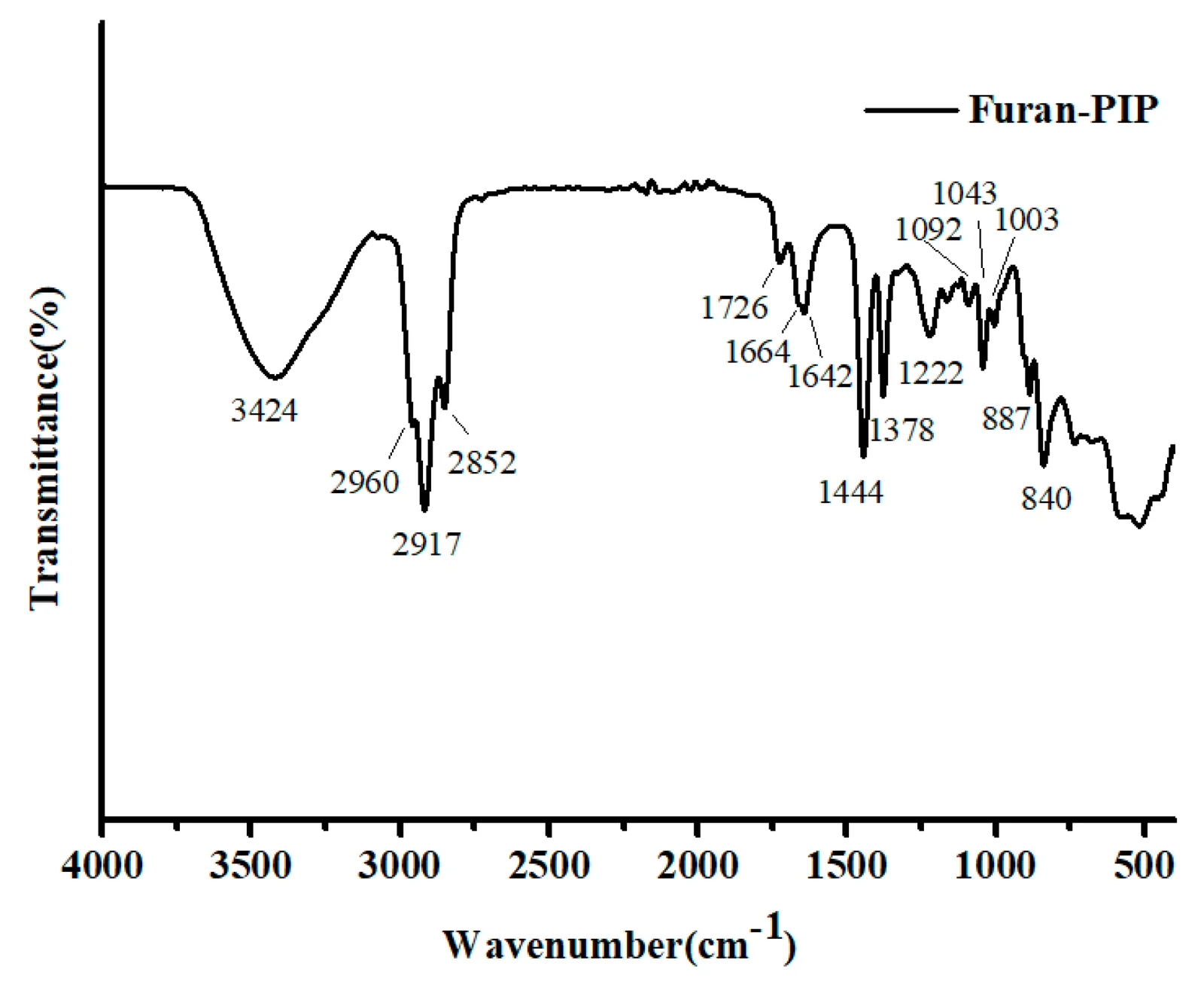
FT-IR spectra for Furan-PIP.

The structural features of the Furan-PIP sample were investigated by FT-IR spectroscopy, which was employed to investigate the dual role of the redox-initiator in the emulsion polymerization of isoprene. Figure 1 presents the spectra of Furan-PIP. with the corresponding functional group assignments listed in Table 1.

The spectrum exhibits characteristic absorptions of the trans-1,4-polyisoprene backbone. The asymmetric stretching vibrations of CH_3_ and CH_2_ are observed at 2960 cm^−1^ and 2917 cm^−1^, respectively, while the symmetric stretching of CH_2_ appears at 2852 cm^−1^. The CH_2_ deformation and symmetric bending of CH_3_ are identified at 1444 cm^−1^ and 1378 cm^−1^, respectively. The C=C stretching vibration of the 1,4-unit is located at 1664 cm^−1^ [31], and the out-of-plane bending vibration of C–H in the –CH=CH– group of the trans-1,4-unit is found at 840 cm^−1^ [20], confirming the predominant trans-1,4 configuration.

In addition to the backbone absorptions, several bands indicative of chemical modifications are present. A broad O–H stretching band at 3424 cm^−1^ and a carbonyl (C=O) stretching band at 1726 cm^−1^ suggest the occurrence of ring-opening reactions of epoxy groups [16,27]. Residual epoxide functionalities are evidenced by the symmetric stretching of the epoxide ring at 1222 cm^−1^ and the C–O–C stretching vibration of the epoxide ring at 1003 cm^−1^ [16,20]. Furthermore, the stretching vibration of the C–O–C bond in the furan ring at 1092 cm^−1^ confirms the formation of furan rings along the polymer backbone [16].

Collectively, the FT-IR data confirm that Furan-PIP retains a trans-1,4-polyisoprene backbone while simultaneously bearing hydroxyl, carbonyl, epoxide, and furan moieties, highlighting the multifunctional reactivity of the redox initiator system and the unique structural characteristics imparted by the concurrent ring-opening, epoxidation, and furanization reactions.

### 3.2. GPC

The molecular weight of Furan-PIP was determined by GPC. The number-average molecular weight ($\bar{M_{n}}$) was 45,892 g/mol with a dispersity (PDI) of 2.362; these are summarized in Table 2, confirming a high-molecular-weight polymer consistent with the proposed emulsion polymerization mechanism.

### 3.3. ^1^H NMR

**Figure 2:**
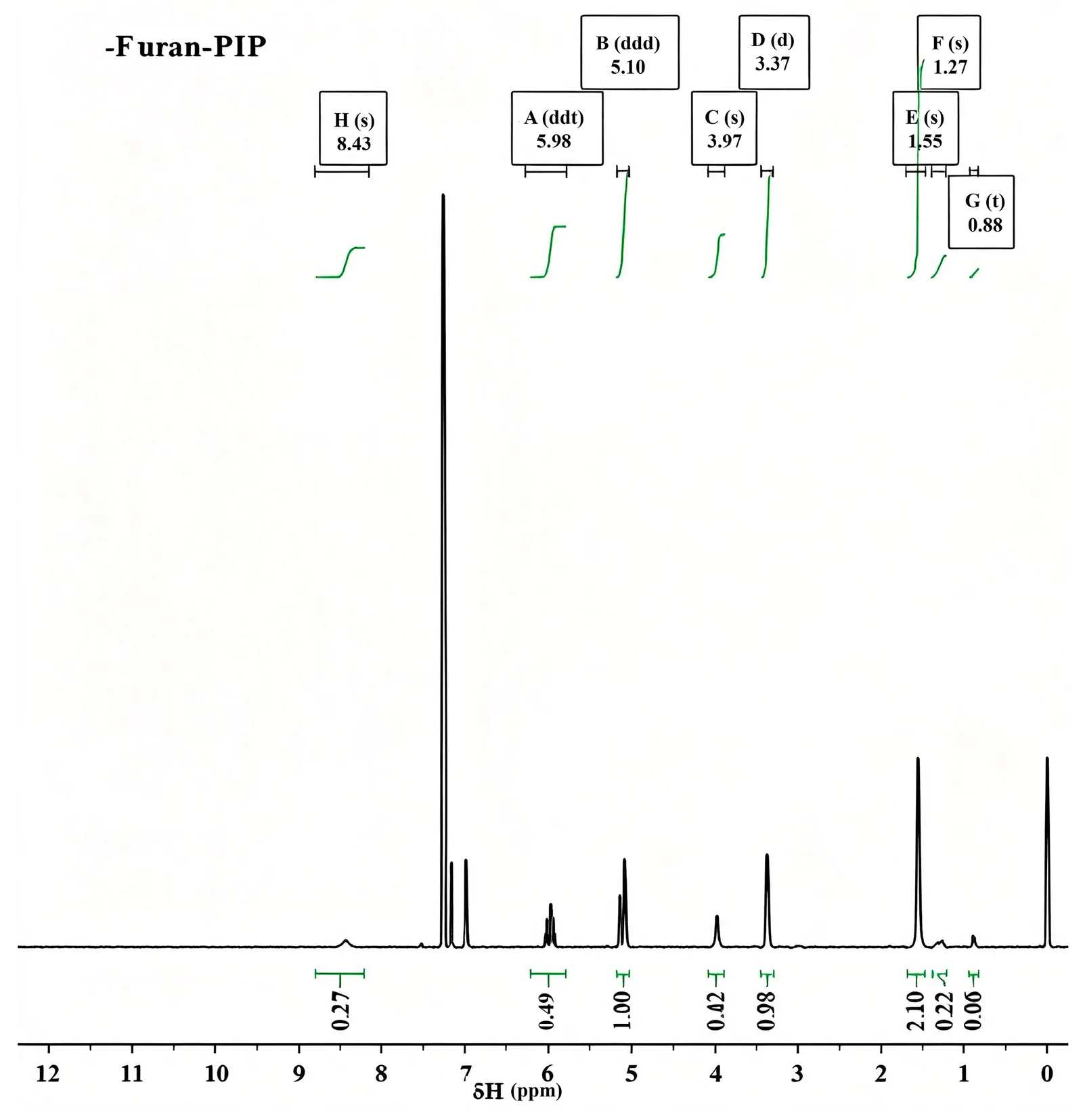
^1^H NMR spectra of Furan-PIP.

The structural features of Furan-PIP were further elucidated by ^1^H NMR spectroscopy, building upon the functional group information obtained from FT-IR analysis. The spectrum is presented in Figure 2, with the corresponding chemical shift assignments summarized in Table 3.

The spectrum exhibits characteristic signals of the trans-1,4-polyisoprene backbone. The methyl protons of the trans-1,4-units appear at 1.55 ppm, while the –CH= protons of the 1,4-units are observed at 5.10 ppm [10,19,30]. Additionally, a signal at 5.98 ppm is attributed to the –CH= of a trisubstituted alkene [32] and the terminal methyl protons appear at 0.88 ppm [33].

Importantly, the spectrum reveals distinct signals corresponding to furan ring functionalities. Resonances at 3.97 ppm and 3.37 ppm are assigned to the protons of the furan ring [16]. Moreover, a characteristic peak at 8.43 ppm is observed, which is attributed to the –OC(O)H proton of a formyloxy group [24]. The concern that the δ 8.43 ppm signal might arise from TEPA–epoxide adducts is mitigated by the mild drying condition (30 °C, vacuum) and the removal of excess TEPA via methanol washing. Hence, the assignment to formyloxy protons is unaffected. In addition, a weak signal at 1.27 ppm is assigned to the –CH3 group of residual epoxy moieties [20,34], indicating that while the ring-opening and furanization reactions have occurred, a small fraction of epoxy groups remains unreacted. The absence of detectable epoxy methine signals, combined with the presence of furan and formyloxy peaks [16,20,34], demonstrates that the majority of epoxy groups underwent ring-opening and furanization reactions. We propose that TBHP not only facilitates epoxidation but also creates conditions conducive to ring-opening of the newly formed oxiranes. The subsequent oxidation of these ring-opened intermediates ultimately leads to the formation of formate esters, accounting for the observed formyloxy protons. Collectively, the ^1^H NMR data confirm that Furan-PIP retains a trans-1,4-polyisoprene backbone and incorporates furan and formyloxy functionalities, alongside a minor amount of residual epoxy groups. These results corroborate the FT-IR findings, providing complementary evidence for the complex microstructure imparted by the multifunctional reactivity of the redox initiator system.

**Table 3:** Functional groups and classification of Furan-PIP for ^1^H NMR.

Functional Group	Chemical Shift (ppm)	References
-OC(O)H of formyloxy proton	8.43	[24]
-CH= of a trisubstituted alkene	5.98	[32]
-CH= of 1,4-unit	5.10	[10,19,30]
Protons of the substituted furan ring	3.97	[16]
Methylene protons adjacent to the furan ring	3.37	
-CH_3_ of trans-1,4-unit	1.55	[10,19,30]
-CH_3_ of the epoxidized isoprene unit	1.27	[20,34]
Terminal -CH_3_	0.88	[33]

Thus, the ^1^H NMR spectrum (Figure 2) exhibits several diagnostic signals that confirm the successful incorporation of furan moieties and ring-opened structures onto the polyisoprene backbone. Specifically, the signals at δ 3.97 and 3.37 ppm are assigned to the protons of the substituted furan ring, consistent with reports in the literature for furan-functionalized polymers. The singlet at δ 8.43 ppm is characteristic of a formyloxy proton (–OC(O)H), indicating the occurrence of ring-opening reactions. Residual trans-1,4 double bonds are identified by the olefinic proton at δ 5.10 ppm. A weak signal at δ 1.27 ppm suggests the presence of a small amount of residual epoxide groups, implying near-complete consumption of epoxy groups during the cascade process. The FT-IR spectrum (Figure 1) further supports these assignments. The absorption band at 1725 cm^−1^ corresponds to the C=O stretching vibration of the formyloxy group, while the bands at 1015 and 760 cm^−1^ are attributed to the furan ring vibrations. The greatly diminished intensity of the characteristic epoxide band at 887 cm^−1^ is consistent with near-complete epoxide conversion, although a minor residual signal remains detectable. Together, the spectroscopic data provide strong qualitative evidence for the formation of furan-functionalized polyisoprene with residual unsaturation and ring-opened segments.

### 3.4. Proposed Reaction Mechanism

Based on the spectroscopic evidence and previous literature on TBHP/TEPA-initiated polymerization of dienes, a plausible cascade mechanism is proposed for the formation of Furan-PIP (Figure S2). In this pathway, TBHP and TEPA generate tert-butoxyl radicals that initiate the radical polymerization of isoprene. Concurrently, the TBHP/TEPA system also promotes the in situ epoxidation of the resulting polyisoprene double bonds. The epoxide groups then undergo ring-opening and furanization reactions, driven by the oxidizing environment and the presence of TEPA. The exact sequence of these transformations remains to be fully elucidated, but the spectroscopic data confirm the coexistence of furan rings, formyloxy groups, and residual double bonds in the final polymer. Further mechanistic studies, including control experiments and kinetic analysis, are underway and will be reported in due course.

### 3.5. DSC

**Figure 3:**
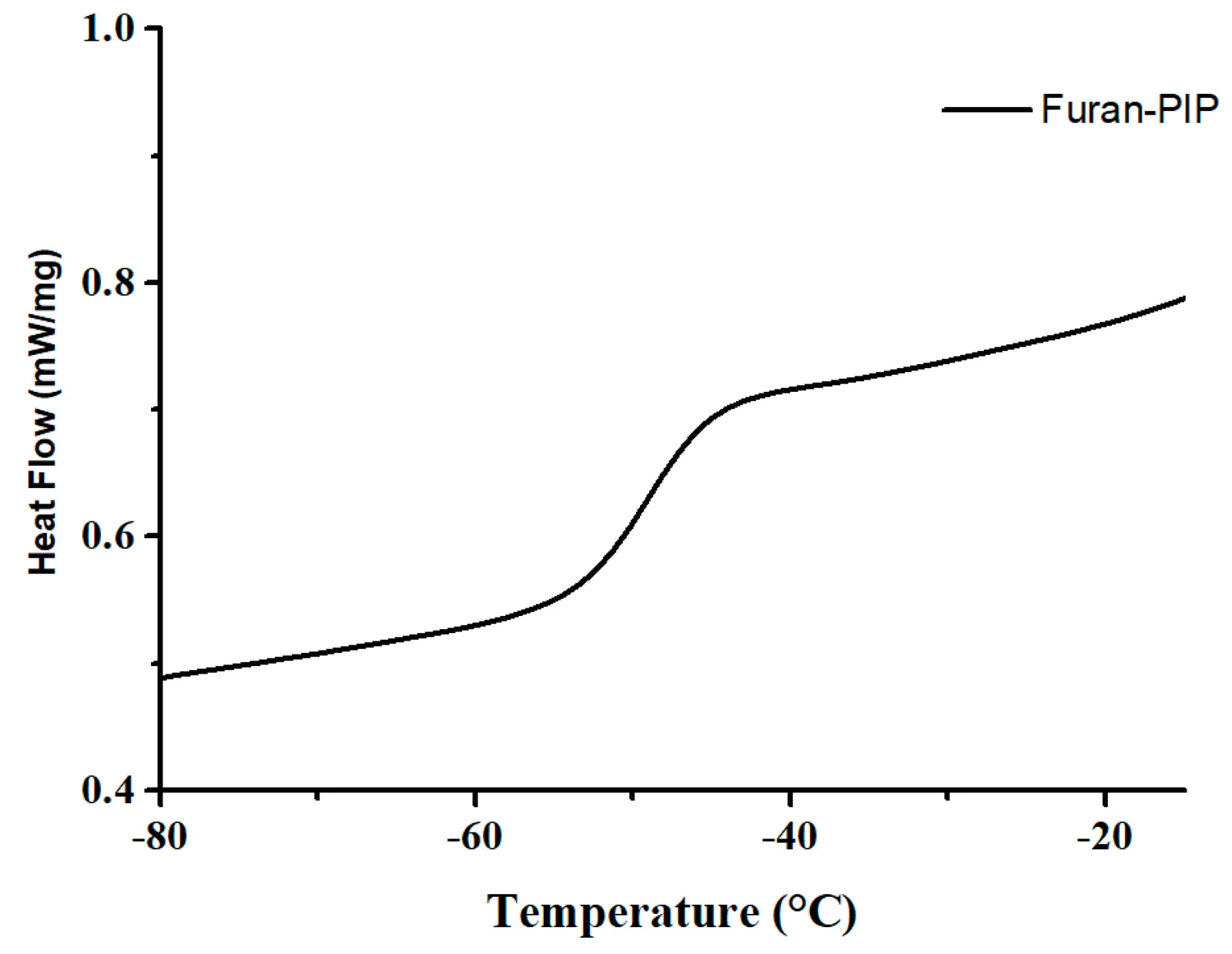
DSC-measured $T_{g}$ of Furan-PIP.

To evaluate the thermal behavior and molecular chain segment mobility of Furan-PIP, differential scanning calorimetry (DSC) was employed to determine its glass transition temperature $T_{g}$. The Tg value reflects the influence of structural factors such as molecular chain flexibility, crosslinking density, and functional group composition. The DSC thermogram of Furan-PIP is presented in Figure 3, and the corresponding Tg parameters are summarized in Table 4.

The DSC thermogram of Furan-PIP is presented in Figure 3. The glass transition temperature (Tg) was determined to be −49.6 °C (midpoint), with onset and endset values of −53.4 °C and −45.6 °C, respectively (Table 3). For comparison, pure cis-1,4-polyisoprene (natural rubber) typically exhibits a Tg of approximately −65 to −70 °C, while pure trans-1,4-polyisoprene has a Tg of approximately −58 to −60 °C. The higher Tg observed for Furan-PIP (−49.6 °C) is consistent with the introduction of rigid furan rings and polar formyloxy groups along the polymer backbone, which restrict chain segmental mobility. This trend is in agreement with previous reports on epoxidized and furan-modified polyisoprenes.

This Tg value is consistent with a furan-modified polyisoprene structure. The introduction of furan rings and ring-opened segments along the polymer backbone may contribute to the observed thermal transition, although a detailed structure–property relationship cannot be established from a single composition.

### 3.6. DMA

To quantify the viscoelastic response of Furan-PIP, dynamic mechanical analysis (DMA) was employed. The storage modulus ($E^{\prime }$), loss modulus ($E^{\prime \prime }$), and damping factor (tan δ) directly reflect the chain mobility, crosslink density, and damping behavior, providing a molecular-level fingerprint of the redox-emulsion architecture.

**Figure 4:**
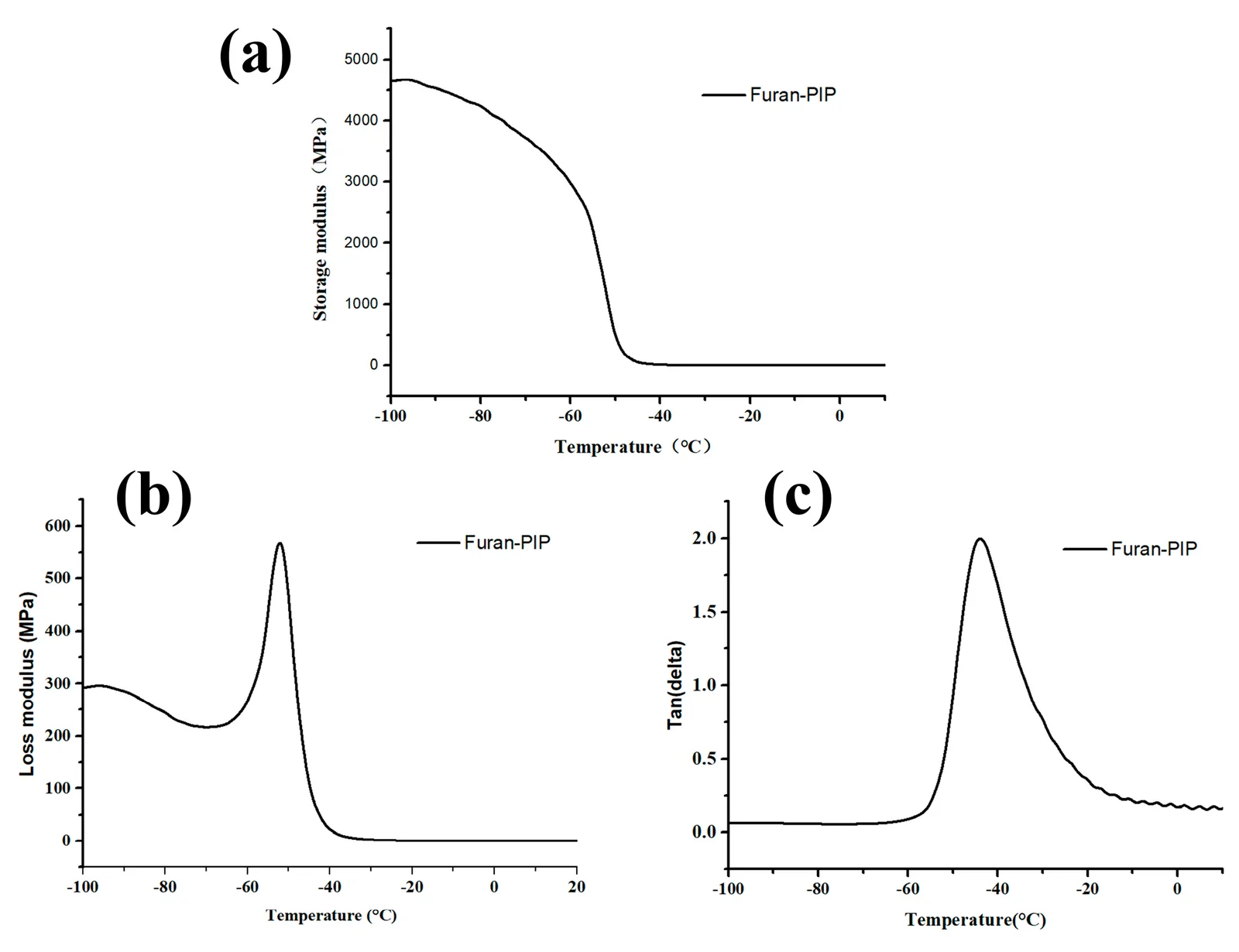
(**a**) Storage modulus ($E^{\prime }$) against temperature for Furan-PIP. (**b**) Loss modulus ($E^{\prime \prime }$) against temperature for Furan-PIP. (**c**) DMA-derived glass transition temperatures of Furan-PIP.

The temperature-dependent viscoelastic responses—$E^{\prime }$, $E^{\prime \prime }$, and tan δ—of Furan-PIP are presented in Figure 4, with the corresponding glass transition temperatures summarized in Table 5.

As shown in Figure 4a, the storage modulus ($E^{\prime }$) of Furan-PIP exhibits a characteristic decrease across the glass–rubber transition region. In contrast to conventional emulsion systems where higher structural uniformity typically elevates E’, the observed modulus reflects the specific structural features of Furan-PIP. The reduction in modulus is primarily attributed to its distinct chemical architecture.

Figure 4b illustrates the loss modulus behavior of Furan-PIP. The $E^{\prime \prime }$ peak, which corresponds to the glass transition, reflects energy dissipation during segmental orientation relaxation. With the epoxy nearly fully consumed and the chemical cross-link density largely fixed, the loss modulus is governed by the chain architecture. Tan δ, the ratio of loss modulus to storage modulus, serves as a measure of damping or energy dissipation. Figure 4c presents the tan δ curve for Furan-PIP, and Table 4 summarizes the characteristic temperatures derived from DMA.

The tan δ peak position is closely related to the glass transition temperature, with the peak maximum observed at –44.02 °C. This value is consistent with the DSC-measured midpoint $T_{g}$ of –49.6 °C (Table 3), with the slight difference attributable to the distinct measurement principles of the two techniques. The DMA results for Furan-PIP are presented in Figure 4. The storage modulus (E′) exhibits a characteristic decrease across the glass-rubber transition region. The loss modulus (E″) peak is observed at −52.12 °C, and the tanδ peak is observed at −44.02 °C (Table 5). This tanδ peak value is consistent with the DSC-measured midpoint Tg of −49.6 °C, with the slight difference attributable to the distinct measurement principles of the two techniques.

The DMA results for Furan-PIP demonstrate that its viscoelastic response is predominantly governed by its chemical composition and architecture. This combination enables effective energy dissipation (manifested as a pronounced tan δ peak) across the glass transition region, resulting in efficient low-temperature damping. These findings corroborate the structural insights obtained from DSC and ^1^H NMR analyses, collectively confirming the complex microstructure imparted by the TBHP/TEPA redox initiator system under the employed synthetic conditions.

### 3.7. TGA

**Figure 5:**
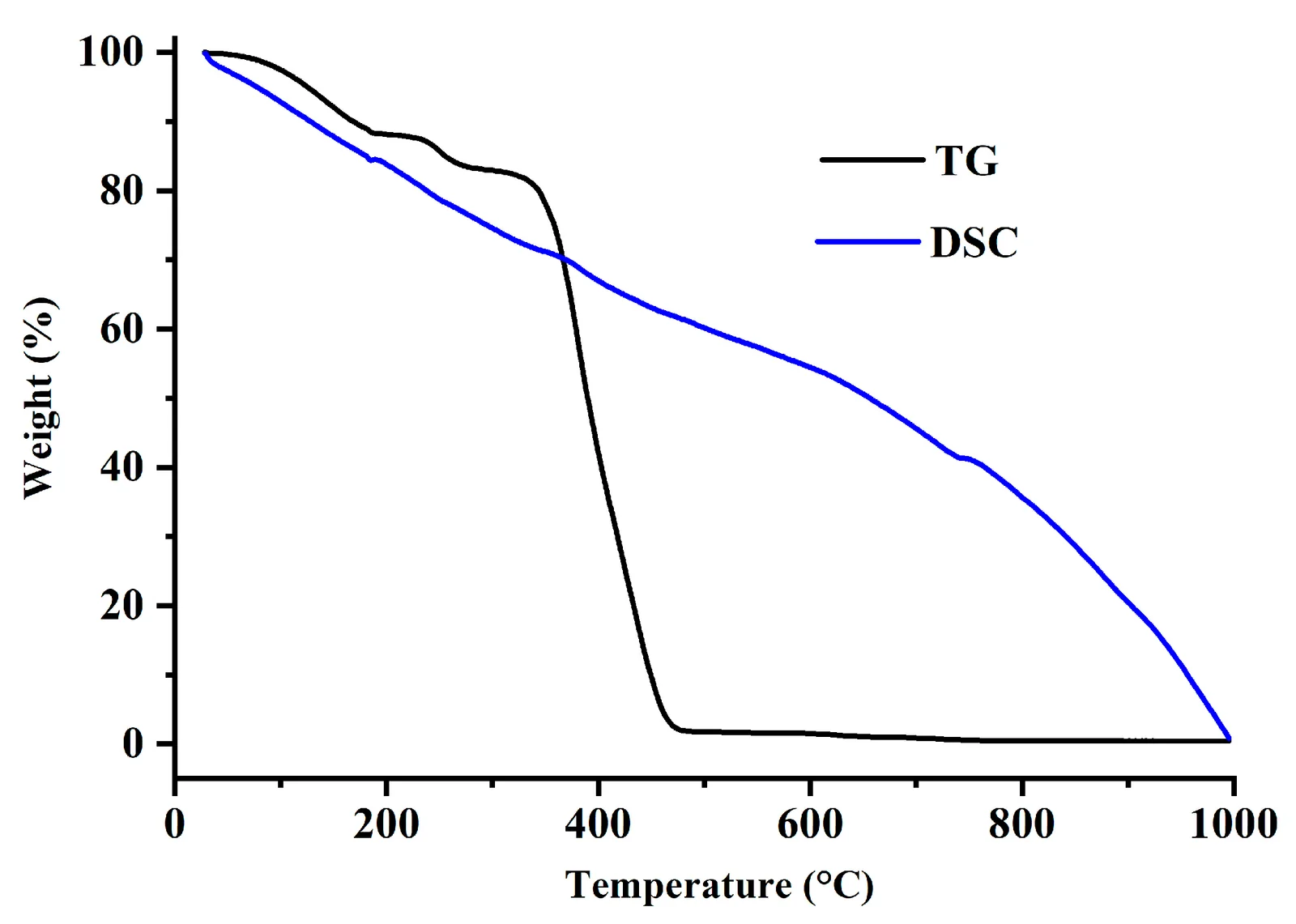
The TGA thermogram of Furan-PIP.

To evaluate the thermal stability and degradation behavior of Furan-PIP, thermogravimetric analysis (TGA) was employed (Figure 5). The thermal decomposition temperature and weight loss curve reflect the influence of structural factors such as molecular chain rigidity, crosslinking density, and functional group composition.

The TGA thermogram of Furan-PIP is presented in Figure 5. The sample exhibits a primary single-stage decomposition profile, characteristic of polyisoprene-based materials. The single-stage decomposition behavior of Furan-PIP is characteristic of polyisoprene-based materials. The observed 312 °C is consistent with a furan-modified polyisoprene structure.

### 3.8. Time-Dependent Antibacterial Performance of Zn(L-Cl)-Furan-PIP Composite

Building on the previously established potent antibacterial activity of the tetranuclear zinc(II) Schiff base complex Zn(L-Cl) against *Staphylococcus aureus*, this study aimed to explore its application as a functional filler within a polymer matrix. To this end, a composite was prepared by uniformly incorporating the Zn(L-Cl) complex into a furan-functionalized polyisoprene (Furan-PIP) rubber matrix, yielding Zn(L-Cl)-Furan-PIP. The design rationale leverages the hydrophobic Furan-PIP matrix as a carrier to enable gradual exposure of the bioactive complex over time, thereby achieving prolonged antibacterial efficacy. The antibacterial efficacy of the Zn(L-Cl)-Furan-PIP composite against S. aureuswas quantitatively evaluated over 24 and 48 h using the standard colony counting method, with the results summarized in Table 6.

**Figure 6:**
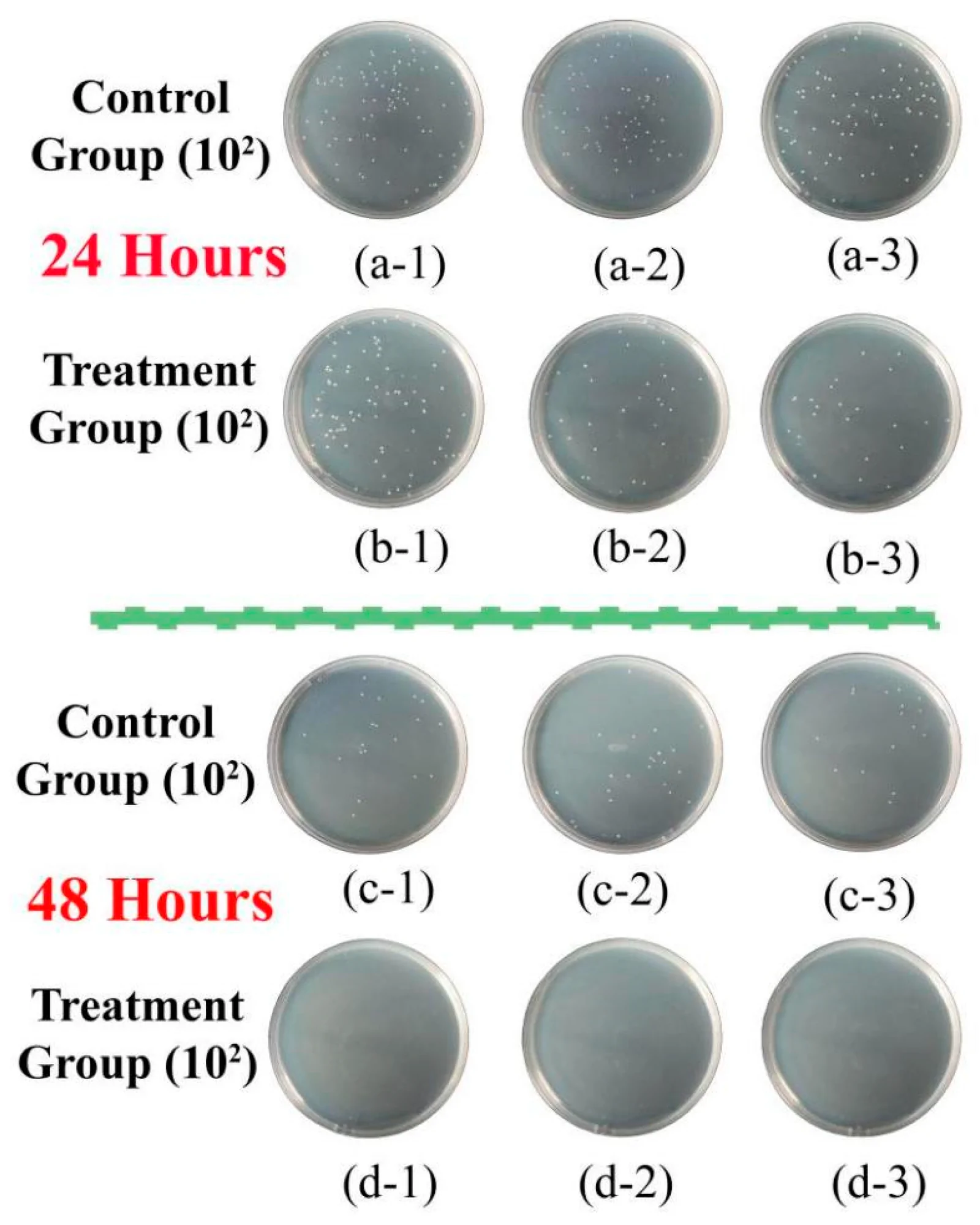
The antibacterial activity over 24 h and 48 h. (**a1**–**a3**) Bacterial colonies in the Control Group at 24 h; (**b1**–**b3**) Bacterial colonies in the Treatment Group at 24 h; (**c1**–**c3**) Bacterial colonies in the Control Group at 48 h; (**d1**–**d3**) Bacterial colonies in the Treatment Group at 48 h. (1, 2, 3 indicate parallel experiments).

After 24 h of contact, the composite exhibited significant antibacterial activity, achieving an inhibition ratio of 44.16% (Table 6). The corresponding bacterial concentration for Zn(L-Cl)-Furan-PIP was determined to be 4.3 × 10^4^ CFU/mL, substantially lower than the 7.7 × 10^4^ CFU/mL measured for the Control-Furan-PIP group. This initial efficacy is visually corroborated in Figure 6, where a clear reduction in *S. aureuscolony* density is observed for the Zn(L-Cl)-Furan-PIP composite group compared to the confluent bacterial lawn present in the Control-Furan-PIP group. This demonstrates the successful initial release of antibacterial agents from the composite matrix.

Remarkably, extending the contact time to 48 h led to a dramatic enhancement of bactericidal efficacy. The inhibition ratio increased to 98.85% (Table 6), with the bacterial load in the test sample reduced to a mere 2.3 × 102 CFU/mL. The potency of this effect necessitated the presentation of colony counts from the undiluted (100) sample, as detailed in Table 6. A high level of bacterial elimination was achieved. The near-absence of colonies in the undiluted Zn(L-Cl)-Furan-PIP samples contrasts with the dense growth in the undiluted control. Furthermore, even at a 10^2^ dilution, the composite-treated group displayed fewer colonies than its corresponding control.

**Figure 7:**
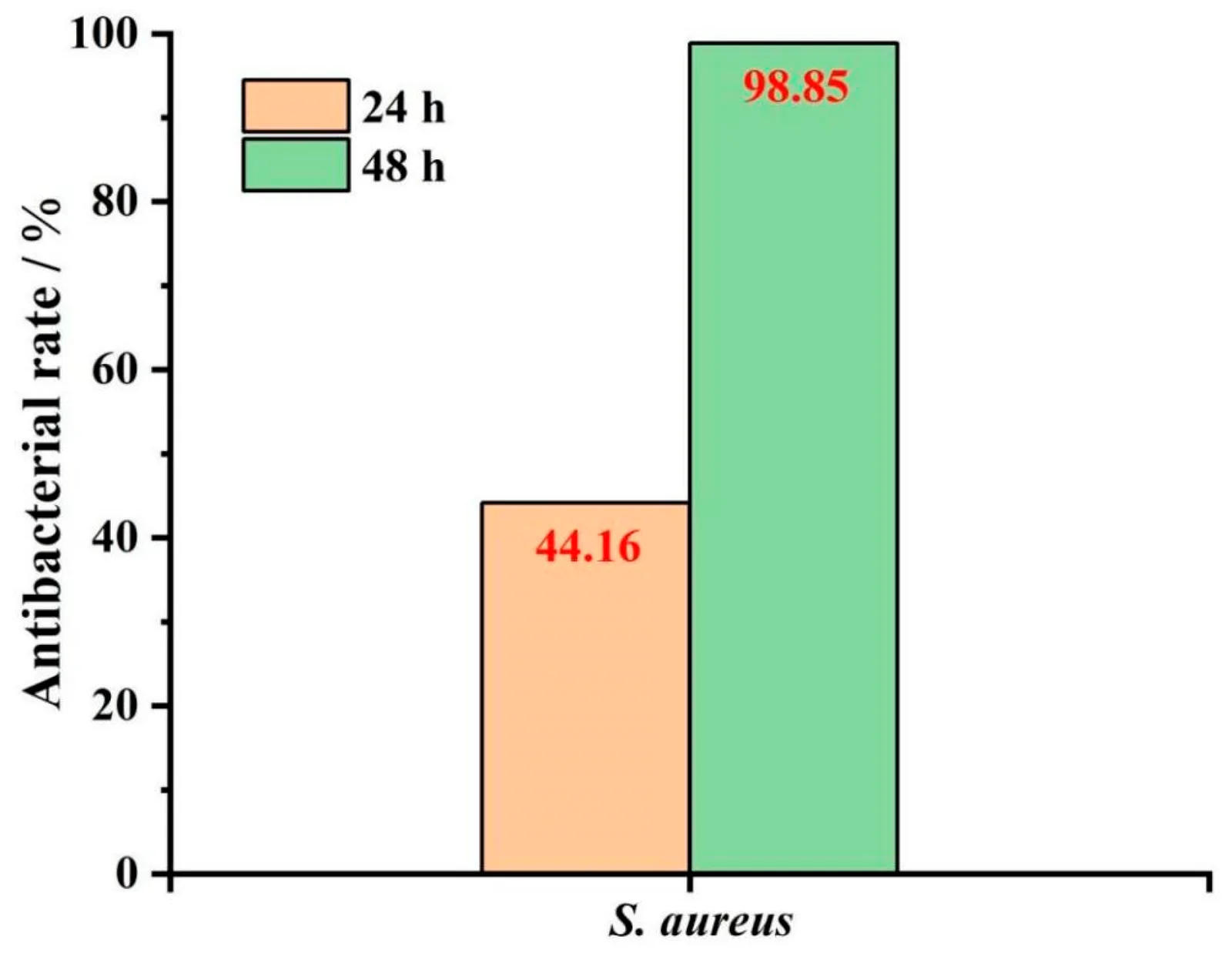
The antibacterial activity over 24 and 48 h, respectively.

The time-dependent escalation of antibacterial efficacy, from 44.16% at 24 h to 98.85% at 48 h, is graphically summarized in Figure 6 and Figure 7. This progressive increase demonstrates a clear time-dependent antibacterial effect. It is noteworthy that the bacterial concentration in the Control-PIP group naturally decreased from 7.7 × 10^4^ CFU/mL to 2.0 × 10^4^ CFU/mL over the same period, a decline attributable to nutrient depletion and accumulation of metabolic waste in the stationary-phase culture. This background reduction in control viability further accentuates the potent and active bactericidal effect of the Zn(L-Cl)-Furan-PIP composite, which achieved high-level bacterial elimination under identical conditions.

The time-dependent antibacterial performance of the Zn(L-Cl)-Furan-PIP composite can be attributed to the intrinsic antimicrobial activity of the Zn(L-Cl) complex itself, combined with its gradual exposure from the polymer matrix. As established in our prior work, the Zn(L-Cl) complex possesses potent antibacterial activity against *S. aureus*, potentially through mechanisms involving membrane disruption and reactive oxygen species (ROS) induction. Within the composite, the Furan-PIP matrix serves as a carrier that physically encapsulates the Zn(L-Cl) complex. The observed increase in antibacterial efficacy from 44.16% at 24 h to 98.85% at 48 h is consistent with a time-dependent exposure mechanism. The initial activity at 24 h may originate from the complex accessible at the surface and near-surface regions, while the enhanced efficacy at 48 h may reflect continued availability of the complex from deeper within the matrix. It should be noted, however, that the present study has not yet systematically measured the release kinetics of the Zn(L-Cl) complex. Therefore, the term “controlled release” is not used here, and the current discussion is confined to describing the observed time-dependent antibacterial efficacy. Future work will focus on establishing quantitative monitoring of the complex release to fully elucidate the release behavior and its correlation with antibacterial performance.

In conclusion, the Zn(L-Cl)-Furan-PIP composite successfully demonstrates the conversion of a molecular antibacterial agent into a material with prolonged antibacterial functionality. The Furan-PIP matrix serves as an effective carrier for the Zn(L-Cl) complex through physical encapsulation, achieving time-dependent antibacterial activity (44.16% at 24 h, reaching 98.85% at 48 h). This antibacterial efficacy can be attributed to the intrinsic antimicrobial mechanism of the Zn(L-Cl) complex itself, as established in our previous work. Our previous study demonstrated that this tetranuclear Zn(II)-Schiff base complex exerts its effects through multiple synergistic pathways: (i) electrostatic attraction between the positively charged zinc complex and anionic teichoic acids in the Gram-positive bacterial cell wall, enriching the complex at the bacterial surface; (ii) the binding of zinc ions to carboxyl groups in the peptidoglycan layer, compromising cell wall integrity; and (iii) the induction of reactive oxygen species (ROS) generation, causing oxidative damage. The time-dependent enhancement of antibacterial activity observed here is consistent with the potent killing capability (>99.99%) of the complex against *S. aureus* reported in our previous work. Nevertheless, it should be noted that the present study has not yet systematically measured the release kinetics of the Zn(L-Cl) complex, nor has it conducted biofilm inhibition assays or cytocompatibility evaluations. Consequently, the current discussion on the antibacterial mechanism is primarily confined to a qualitative “structure–release–exposure” framework. Future work will focus on establishing quantitative monitoring of the complex release, incorporating biofilm models and mammalian cell cytotoxicity tests, in order to more comprehensively elucidate the antibacterial mechanism and assess the feasibility of biomedical applications.

## 4. Conclusions

In summary, a furan-functionalized polyisoprene (Furan-PIP) was successfully synthesized via a one-pot, redox-initiated cascade approach employing the TBHP/TEPA initiating system. The polymerization of isoprene proceeded through radical emulsion polymerization, coupled with in situ epoxidation, ring-opening, and furanization reactions. FT-IR and ^1^H NMR spectroscopy qualitatively confirmed the formation of the desired furan and formyloxy functionalities, while the nearly complete disappearance of epoxide signals indicated the near-complete consumption of epoxy groups. GPC analysis further confirmed the high molecular weight of the polymer ($\bar{M_{n}}$ = 45,892 g/mol, PDI = 2.362). Thermal analysis revealed a Tg of −49.6 °C and a single-stage decomposition behavior, consistent with a furan-modified polyisoprene structure.

An antibacterial composite was fabricated by physically encapsulating a pre-synthesized zinc complex Zn(L-Cl)—previously reported by our group to exhibit antibacterial activity into the Furan-PIP matrix. The composite exhibited time-dependent antibacterial activity against *Staphylococcus aureus* over 48 h, highlighting its potential as a long-lasting antibacterial material.

This work provides a simple and efficient synthetic route to furan-functionalized elastomers and demonstrates their applicability as carriers for antibacterial agents. Future studies will focus on elucidating the detailed reaction mechanism and exploring the structure–property relationships of these novel materials.

## Figures and Tables

**Table 1:** Functional groups and classification of compounds for Furan-PIP.

Functional Group	Wavenumber (cm^−1^)	References
O-H stretching	3424	[16,27]
CH_3_ asymmetric stretching	2960	[29,30]
CH_2_ asymmetric stretching	2917	
CH_2_ symmetric stretching	2852	
(C=O) stretching	1726	[16,27]
C=C stretching vibration of 1,4-unit	1664	[31]
C=C stretching vibration	1642	[29]
CH_2_ deformation	1444	
CH_3_ symmetric	1378	
Symmetric stretching of the epoxide ring	1222	[16,20]
Stretching vibration of the C-O-C bond in the furan ring	1092	[16]
Stretching or wagging vibration of CH3C=C in trans-1,4-unit	1043	[31]
Stretching vibration of C–C	1003	[29]
Stretching vibration of epoxide C-O-C of epoxide ring	887	[20]
Out-of-plane bending vibration of C–H in the –CH=CH– group of trans-1,4-unit	840	[31]

**Table 2:** Average molecular weight and PDI of Furan-PIP.

Formulation	$\bar{M_{n}}$	$\bar{M_{w}}$	PDI
Furan-PIP	45,892 g/mol	108,415 g/mol	2.362

**Table 4:** Comparative Onset, Mid-point and Endset $T_{g}$ of Furan-PIP.

**Formulation**	Onset ($T_{g}$/°C)	Midpoint ($T_{g}$/°C)	Endset ($T_{g}$/°C)
Furan-PIP	−53.4	−49.6	−45.6

**Table 5:** DMA-derived glass transition temperatures of Furan-PIP.

Formulation	Storage Modulus Onset/°C	Loss Modulus Peak/°C	tan δ Peak/°C
Furan-PIP	–54.27	–52.12	–44.02

**Table 6:** The experimental data on the antibacterial activity over 24 h and 48 h.

Group	Colony-Forming Unit (CFU)	Dilution Ratio	Concentration (CFU/mL)	Antibacterial Ratio
24 h				
Control Group-1	89	10^2^	7.7 × 10^4^	/
Control Group-2	65	10^2^		
Control Group-3	77	10^2^		
Zn(L-Cl)-Furan-PIP-1	78	10^2^	4.3 × 10^4^	44.16%
Zn(L-Cl)-Furan-PIP-2	28	10^2^		
Zn(L-Cl)-Furan-PIP-3	23	10^2^		
48 h				
Control Group-1	20	10^2^	2 × 10^4^	/
Control Group-2	24	10^2^		
Control Group-3	16	10^2^		
Zn(L-Cl)-Furan-PIP-1	20	10^0^	2.3 × 10^2^	98.85%
Zn(L-Cl)-Furan-PIP-2	19	10^0^		
Zn(L-Cl)-Furan-PIP-3	30	10^0^		

## Data Availability

The data supporting the findings of this study are available within the article and its Supplementary Materials.

## Supplementary Materials

The following supporting information can be downloaded at: https://www.mdpi.com/article/10.3390/polym18172086/s1, Figure S1: SEM elemental mapping image showing the spatial distribution of zinc (Zn) on the sample surface; Figure S2. Mechanism of epoxide consumption via ring-opening–furanization cascade in Furan-PIP. Table S1: Reagents, consumables, and bacterial strain; Table S2: Instruments and equipment; Table S3: Parameter for DMA.
