# Supplementary material for: A Redox-Initiated Cascade Approach to Furan-Functionalized Polyisoprene with Time-Dependent Antibacterial Activity
Source: Polymers (Basel). 2026 Aug 28;18(17):2086. doi: 10.3390/polym18172086 (PMC13567968; doi:10.3390/polym18172086)
Supplement: Supplementary file 1 [file polymers-18-02086-s001.zip › polymers-4410525-supplementary.pdf]

A Redox-Initiated Cascade Approach to Furan-Functionalized Polyisoprene with Time-Dependent Antibacterial Activity

Cui-Cui Wang [1,2,†] and Jin-Hua Wang [1,*,†]

[a]*School of pharmacy, Shandong Provincial Engineering Research Center of Novel Pharmaceutical Excipients, Sustained and Controlled Release Preparations, Dezhou University, Dezhou, Shandong, 253015, PR China*

[b]*University Kuala Lumpur, Malaysian Institute of Chemical and Engineering Technology, Lot 1988 Kawasan Perindustrian Bandar Vendor, Taboh Naning, 78000, Alor Gajah, Melaka, Malaysia*

[*]Corresponding authors. E-mail: wangjinhua1071@163.com (Jin Hua Wang)

[†]These authors contributed equally to this work

1. Main Reagents and Equipment

1.1 Reagents, Consumables, and Bacterial Strain

Table S1. Reagents, Consumables, and Bacterial Strain

Reagent/Strain	Purities	Supplier	Location
Isoprene	99%	Tokyo Chemical Industry Co., Ltd	Tokyo, Japan
Dihexyl sodium sulfosuccinate	98%	Meryer Chemical Technology Co., Ltd.	Shanghai, China
tert-butyl hydroperoxide(TBHP, 70% in water)	70% in water	Tokyo Chemical Industry Co., Ltd.	Tokyo, Japan
Tetraethylenepentamine (TEPA)	95%	Tokyo Chemical Industry Co., Ltd.	Tokyo, Japan
Methanol	99%	ROSEN Technologies (Beijing) Co., Ltd.	Beijing, China

Sodium hydroxide (NaOH)	98%	ROSEN Technologies (Beijing) Co., Ltd.	Beijing, China
LB Broth Medium	BR	Qingdao Haibo Biotechnology	Qingdao, China
Agar Powder	BR	Qingdao Haibo Biotechnology	Qingdao, China
Staphylococcus aureus	Standard strain	Guangdong Microbial Culture Collection Center	Guangdong, China

1.2 Instruments and Equipment

**Table S2.** Instruments and Equipment			
Equipment	Model	Supplier	Location
High-Pressure Steam Sterilizer	BXM-110FI	Shanghai Boxun	Shanghai, China
Clean Bench	BJ-2CD	Shanghai Boxun	Shanghai, China
Constant Temperature Shaker	BXYC-LX3400	Shanghai Boxun	Shanghai, China
Analytical Electronic Balance	FA2204N	Shanghai Jinghua (Jinghai)	Shanghai, China
Multifunction Microplate Reader	BioTek Synergy HTX	Agilent	Winchester,United States
Automatic Colony Counter	SCAN1200	Interscience	Puycapel, France

2. General method

2.1 Characterization of redox-initiated emulsion polymerization of Furan-PIP Composites

The chemical structures of the Furan-PIP Composites were analyzed using a Thermo Fisher Nicolet iS50 FTIR spectrometer (USA) equipped with an ATR accessory. The spectra were collected in ATR mode at a resolution of 4 cm^{-1} with 32 scans. ^1H NMR spectra was recorded on a Bruker Ultra Shield 400 MHz spectrometer (Germany) using $CDCl_3$ as the solvent. Chemical shifts are reported in parts per million (ppm) and are referenced to tetramethylsilane (TMS).

DMA was performed on a TA Instruments DMA 850 (USA). Test specimens measuring 6 cm × 1.2 cm were prepared and analyzed in tension mode. The detailed test parameters are summarized in Table S2.

Table S3. Parameter for DMA analysis

**Parameter**	**Setting**
Temperature	-100°C to room temperature
Heating/Cooling rate	2°C/min
Atmosphere	Liquid nitrogen
Tension mode	10 Hz

The glass transition temperature (Tg) of the samples was determined using a DSC (NETZSCH DSC 200 F3, Germany). The sample was cooled down to -100°C with a cooling rate of 20°C /min. Then, the sample was then heated from -100°C to 50°C with a heating rate of 20°C/min under nitrogen gas.

TGA is performed to determine the thermal decomposition of the samples. 10 mg of sample is approximately heated in a TGA at temperatures ranging from 35 ℃ to 850 ℃. With a heating rate of 10°C /min, under a nitrogen flow rate of 20 ml/min. The degradation temperature curves of the samples are recorded and plotted as a function of time. The residual weight (TG) was evaluated.

2.2 Antibacterial Performance of Zn(L-Cl)/Furan-PIP Composites

The experimental procedure was conducted as follows. A single colony of Staphylococcus aureus was picked from a solid agar plate using a sterile inoculation loop and inoculated into a sterile test tube containing 3-5 mL of liquid LB medium. The inoculated tube was then incubated in a constant temperature shaker at 37°C with

agitation at 200-250 rpm for 12-16 hours (typically overnight) until the bacterial suspension reached turbidity, corresponding to an OD600 of approximately 2.0-4.0. This primary culture was diluted with phosphate-buffered saline (PBS) to achieve a concentration of 10^4 CFU/mL. A predetermined mass of the test sample was added to aliquots of this diluted bacterial suspension to attain a final sample concentration of 5 mg/mL. One set of these sample-bacteria mixtures was incubated with shaking at 37°C for 24 hours, while a parallel set was incubated under identical conditions for 48 hours. Following incubation, the bacterial mixtures were subjected to serial ten-fold dilution: a series of centrifuge tubes (labeled 1 to n) were prepared, each containing 900 μL of physiological saline. A volume of 100 μL from the original mixture was added to tube 1 and mixed thoroughly. Subsequently, 100 μL from tube 1 was transferred to tube 2, and this dilution process was repeated sequentially. From an appropriately diluted tube, 100 μL was spread onto fresh LB agar plates. All plates were incubated at 37°C for 18 hours, after which they were photographed for colony enumeration. The antibacterial rate was calculated based on colony counts from a selected appropriate dilution for each test group, with reference to the national standard GBT 21510-2008 (Test method of antibacterial performance for nano-inorganic materials).

3. Scanning electron microscopy (SEM) elemental mapping image

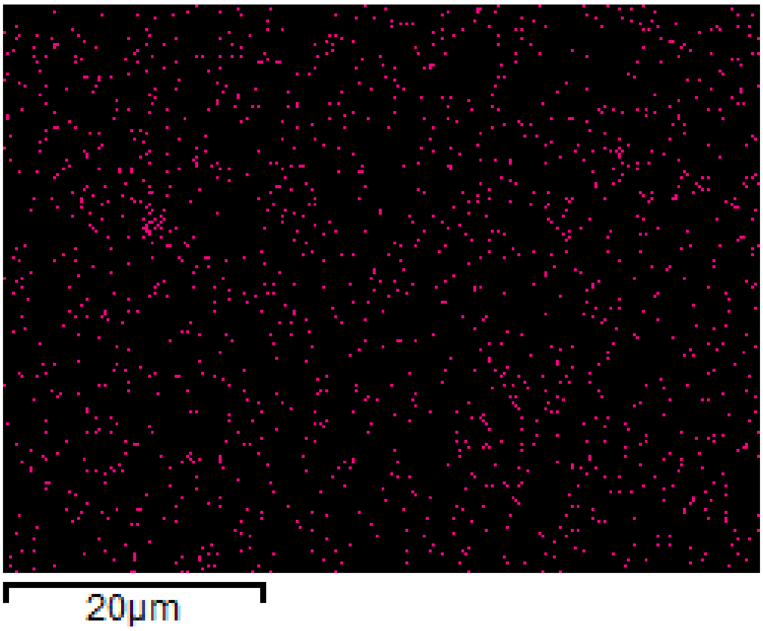

Figure S1. SEM elemental mapping image showing the spatial distribution of zinc

(Zn) on the sample surface.

4. Proposed reaction mechanism

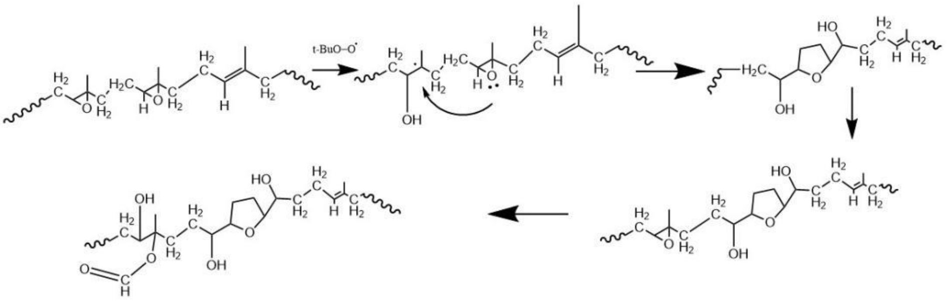

Figure S2. Mechanism of epoxide consumption via ring-opening–furanization cascade in Furan-PIP
